# Time conditioning for arbitrary contrast phase generation in interventional computed tomography

**DOI:** 10.1088/1361-6560/ad46dd

**Published:** 2024-05-20

**Authors:** Mark A Pinnock, Yipeng Hu, Steve Bandula, Dean C Barratt

**Affiliations:** 1 Centre for Medical Image Computing, University College London, London, United Kingdom; 2 Wellcome/EPSRC Centre for Interventional and Surgical Sciences, University College London, London, United Kingdom; 3 Centre for Medical Imaging, Division of Medicine, University College London, London, United Kingdom; 4 Department of Interventional Radiology, University College London Hospitals NHS Foundation Trust, London, United Kingdom

**Keywords:** computed tomography, contrast enhancement, convolutional neural network, interventional radiology, deep learning

## Abstract

Minimally invasive ablation techniques for renal cancer are becoming more popular due to their low complication rate and rapid recovery period. Despite excellent visualisation, one drawback of the use of computed tomography (CT) in these procedures is the requirement for iodine-based contrast agents, which are associated with adverse reactions and require a higher x-ray dose. The purpose of this work is to examine the use of time information to generate synthetic contrast enhanced images at arbitrary points after contrast agent injection from non-contrast CT images acquired during renal cryoablation cases. To achieve this, we propose a new method of conditioning generative adversarial networks with normalised time stamps and demonstrate that the use of a HyperNetwork is feasible for this task, generating images of competitive quality compared to standard generative modelling techniques. We also show that reducing the receptive field can help tackle challenges in interventional CT data, offering significantly better image quality as well as better performance when generating images for a downstream segmentation task. Lastly, we show that all proposed models are robust enough to perform inference on unseen intra-procedural data, while also improving needle artefacts and generalising contrast enhancement to other clinically relevant regions and features.

## 1. Introduction

While surgical techniques such as radical or partial nephrectomy are the mainstay of treatment for renal cell carcinoma, minimally invasive, nephron-sparing procedures offer a lower rate of complications and more rapid recovery (Mues and Landman 2010). One of these techniques is cryoablation, which uses needles inserted in and around the tumour to cause cell death through the freeze-thaw effect (Uppot *et al* 2009). Interventional computed tomography (iCT) provides excellent, low cost visualisation of the kidney, tumour and surrounding anatomy during procedures such as cryoablation (Permpongkosol *et al* 2006), but requires intravenous iodinated radiocontrast agents (RCAs) for anatomical contrast, which are not without their drawbacks: as well as the required increase in radiation dose (Sahbaee *et al* 2017) and the short washout period requiring repeated dosing, RCAs have also been associated with allergic and nephrotoxic complications (Bottinor *et al* 2013), making it essential to limit their use where possible.

In our institution, a typical cryotherapy procedure starts with a non-contrast-enhanced (NCE) series, followed by patient re-positioning as required. After injection of RCA, two series at 35 and 80 seconds can be acquired, corresponding to corticomedullary enhancement (CME) and nephrogenic enhancement (NGE) phases. The enhancement seen in these series fades over the subsequent minutes as RCA is eliminated from the nephrons and enters the collecting system (Seager *et al* 2020). Supplemental figure S1 (online) illustrates how this process occurs and how the contrast enhancement pattern changes over the course of a procedure.

RCA dosage can be reduced by using low tube voltages (Nakaura *et al* 2011) and iterative reconstruction algorithms (Feng *et al* 2018), but a current research question is whether the use of RCAs (and subsequent adverse events) can be eliminated entirely using computational techniques. Histogram-based techniques (Al-Ameen *et al* 2015) optimise the overall contrast of an image but do not account for the local effect of contrast agents. Level set-based (Bitter *et al* 2006) and oriented filter-based methods (Mukherjee and Acton 2015) offer blood vessel enhancement, but require hand-crafted filters and do not generalise well to other clinically relevant structures.

Convolutional neural networks (CNNs) have become ubiquitous in medical image analysis, and have also recently been applied to contrast enhancement, for instance reducing gadolinium dosage in brain magnetic resonance imaging (Gong *et al* 2018, Kleesiek *et al* 2019, Montalt-Tordera *et al* 2021) or enhancing liver tumours with respect to background tissue (Zheng *et al* 2021). Deep learning has also been used to convert contrast-enhanced CT to non-contrast-enhanced CT, using both CNNs (Sumida *et al* 2019) and generative adversarial networks (GANs) (Sandfort *et al* 2019, Li *et al* 2021), while the latter has been used to create contrast in magnetic resonance flow imaging (Bustamante *et al* 2021).

Recently there has been a growing interest in generating synthetic contrast-enhanced (sCE) CT images to avoid the use of RCAs entirely (Choi *et al* 2021, Kim *et al* 2021). Liu *et al* developed a two-stage GAN (generating first coarse and then fine detail) (Liu *et al* 2020) while Chandrashekar *et al* and Xie *et al* opted to use CycleGAN, a GAN architecture designed for style transfer in unpaired data (Chandrashekar *et al* 2020, Xie *et al* 2021). While these studies show that it is possible to generate sCE images, they perform this only for early arterial enhancement, whereas the optimal phase depends on the application. In renal CT, NGE phase series have the greatest sensitivity for detecting renal tumours, whereas CME series can improve accuracy and lower false-positive rates (Yu *et al* 2017).

**Figure 1.**
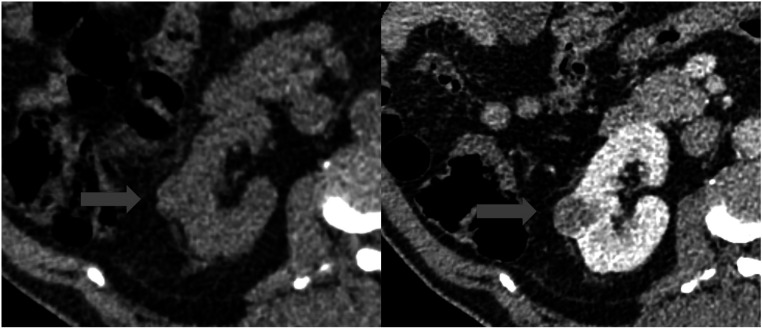
Left: NCE image with barely perceptible tumour (red arrow). Right: NGE phase image clearly showing location of tumour.

Given that contrast enhancement patterns are generated by processes far below the resolution of clinical scanners, the question as to what exactly is causing CNNs to learn these contrast enhancement patterns remains unknown and is not readily explainable with biophysical and biophysiological mechanisms. One potential mechanism is illustrated in figure 1. The left-hand image shows a tumour (red arrow) that is challenging to distinguish from the surrounding tissue with the human eye, while the right-hand NGE phase image is much clearer. However, after segmenting the tumour and kidney in the NCE image using the overlayed NGE image, we find that the mean intensity in the tumour region on the NCE images is 35 HU versus 44 HU in the surrounding tissue (significant on t-test, *p* < 1*e* − 9), indicating that there is potentially useful information in the NCE volumes not easily visible to the human eye.

While the focus of previous works has to been to determine whether a neural network can be trained to generate sCE images (including both CME and NGE phases), it is unknown whether it can generalise the mapping to arbitrary time-points after injection to allow us to select a continuous range of sCE patterns. As well as wider applications outside of the medical imaging domain, the generated phase can be tuned as a hyper-parameter for a variety of downstream medical imaging tasks, for instance allowing more flexible visualisation of relevant regions of interest (ROIs), augmentation of scarce clinical data, or determining the optimal sCE pattern for segmentation.

HyperNetworks (Ha *et al* 2017) have been proposed as a means to generate weights for a larger network using a smaller network, typically using a much smaller number of trainable parameters. Whereas the standard training process updates the convolutional kernel weights directly by backpropagation, a HyperNetwork consisting of a few fully connected layers generates the weights used in the convolutional layers of the main network, and is itself trained by backpropagation. In order to train a neural network to generate more than one contrast phase, it is necessary to condition the network on the phase required. Typically with GANs, this is achieved by concatenating the class information to the feature maps at some point in the generator and discriminator architecture (Mehdi and Osindero 2014). This has been shown to be effective and can allow switching of the contrast phase at inference(Pinnock *et al* 2023), acting as a baseline for interpolating contrast phases. Interpolating the weights of a neural network representing an image has been shown to be more effective than interpolating the image pixels themselves (Klocek *et al* 2019), leading to the primary hypothesis of this paper: whether conditioning the convolutional kernels via a HyperNetwork is more effective for conditional style transfer applications such as synthetic contrast enhancement, particularly when generalising to phases unseen during training.

Medical images have substantial anatomical information that may be of use in contrast enhancement. While features in natural images may appear in multiple regions of the image (trees or animals are not confined to one location, for instance, and may appear in different regions relative to each other), the spatial relationships between anatomical features are typically much more conserved, implying that a full receptive field should be beneficial. However, volumes from iCT data are challenging to pair, becoming less well-aligned over time as the subject is repositioned and organs move. A preliminary experiment investigating sCE performance indicated that decreasing the receptive field size could help tackle this problem when combined with a weighted loss (detailed in Methods), thereby improving convergence, image quality and accuracy of the generated contrast. Patch-based techniques have also been proposed for style transfer in natural images and are purported to preserve the geometrical structure of the input image (Huang and Chien 2020). Therefore, a secondary hypothesis to be investigated is that operating on image patches can improve performance in sCE applications.

The overall aim of this pilot study was to assess the feasibility of modelling the full contrast enhancement process. We first focus on evaluating two different techniques of conditioning neural networks to generate CME and NGE contrast phases, while investigating the primary and secondary hypothesis outlined above. We then generalise this process to generate sCE images at arbitrary time points between CME and NGE and explore the effect of tuning the generated phase on a specific downstream task.

To the best of our knowledge, this is the first application of HyperNetworks for synthetic contrast enhancement by incorporating phase information as a hyper-parameter, as well as the first to attempt to generalise the sCE process to a continuous set of time-points during the contrast enhancement process. While iCT data is hard to acquire, we show significance on the largest dataset that we have found so far in the literature. The proposed research questions are:

1. Does the use of a HyperNetwork improve the ability of a neural network in generating variable contrast phases?
2. Does the receptive field of neural networks affect the ability to generate realistic sCE?
3. Can interpolating the sCE process generate an optimal phase for a segmentation task?

To answer the above questions, we have made the following contributions: (1) we evaluate a modified Pix2Pix (Isola *et al* 2017) architecture, chosen as a well-established technique in the style transfer domain, that can switch the contrast phase of generated images as a baseline; (2) we test an additional modification to Pix2Pix by using a HyperNetwork to generate convolutional kernels by considering phase information as a hyper-parameter; (3) we compare the performance of the proposed models trained on the entire image versus patches; (4). We assess the performance of these methods in generating an optimal interpolated sCE phase for a U-Net (Ronneberger *et al* 2015) trained to perform segmentation on renal tumours; (5) we train the models on a clinical interventional dataset, and test the models’ robustness during inference on previously unseen, challenging intra- and post-procedural images. We show that the benefits of this technique go beyond enhancement of vascular regions and could be developed for use in applications such as de-noising and artefact removal.

## 2. Methods

### 2.1. Baseline network architecture

The proposed technique is compared against a baseline method for switching contrast phases. Owing to its ability to generate intense sCE images in multi-phase applications (Pinnock *et al* 2023), a modified Pix2Pix is used as the baseline technique, where the generator *f*_*θ*_ is based on a 3D U-Net (Çiçek *et al* 2016) using instance normalisation. This network takes source images **x**, noise in the form of dropout **z** and phase information *t* as input, and outputs the predicted target images ${\hat{{\bf{y}}}}_{t}$ for the specified phase. The Pix2Pix is conditioned on the phase by concatenating the scalar phase information (1 representing CME, 2 representing NGE) to the feature maps

\begin{eqnarray*}{\hat{{\bf{y}}}}_{t}={f}_{\theta }({\bf{x}},{\bf{z}},t).\end{eqnarray*}

Each down-sampling block in the encoder uses one convolutional layer, while up-sampling decoder blocks use a transpose convolution and then a standard convolution after concatenation with the skip layer. The number of channels in each down-sampling block is doubled in successive layers while the converse is true in the encoder. 50% dropout is applied to the first three layers of the decoder at training and inference to add noise. The discriminator *g*_*ϕ*_ is a 3D version of the PatchGAN network described in the same paper, and is trained on both the real target images **y**_*t*_ and generated target images *f*_*θ*_(**x**, **z**, *t*). As this architecture is well-validated in this domain, we omit the details for brevity and refer the reader to the original paper (Isola *et al* 2017).

As well as the adversarial component, the Pix2Pix loss adds a supervisory term provided to the generator via the *L* ^1^-norm between the generated and target images. To improve convergence based on observations from preliminary work, we split this term ${{ \mathcal L }}_{\mathrm{ROI}}$ into a foreground ${{ \mathcal L }}_{F}$ and background ${{ \mathcal L }}_{B}$ loss using segmentation masks of the arterial vasculature, kidneys and tumour, weighted by a hyperparameter *μ*. The masks are represented below by the indicator function **1** for foreground **1**_*F*_ and background **1**_*B*_.

\begin{eqnarray*}\begin{array}{rcl}{{ \mathcal L }}_{F} &amp; = &amp; {\left|{{\bf{1}}}_{F}\,[{{\bf{y}}}_{t}-{f}_{{\boldsymbol{\theta }}}\,({\bf{x}},{\bf{z}},t)]\right|}_{1}\\ {{ \mathcal L }}_{B} &amp; = &amp; {\left|{{\bf{1}}}_{B}\,[{{\bf{y}}}_{t}-{f}_{{\boldsymbol{\theta }}}\,({\bf{x}},{\bf{z}},t)]\right|}_{1}\\ {{ \mathcal L }}_{\mathrm{ROI}} &amp; = &amp; \mu {{ \mathcal L }}_{F}+(1-\mu )\,{{ \mathcal L }}_{B}.\end{array}\end{eqnarray*}

This term is added to the binary cross-entropy loss below, weighted by hyperparameter *λ*:

\begin{eqnarray*}\begin{array}{rcl}{{ \mathcal L }}_{D} &amp; = &amp; -{{\mathbb{E}}}_{{\bf{y}}}\{\mathrm{ln}\,[{g}_{{\boldsymbol{\phi }}}\,({{\bf{y}}}_{t},t)]\}-{{\mathbb{E}}}_{{\bf{x}},{\bf{z}}}\{\mathrm{ln}\,[1-{g}_{{\boldsymbol{\phi }}}\,({f}_{{\boldsymbol{\theta }}}\,({\bf{x}},{\bf{z}},t),t)]\}\\ {{ \mathcal L }}_{G} &amp; = &amp; -{{\mathbb{E}}}_{{\bf{x}},{\bf{z}}}\{\mathrm{ln}\,[{g}_{{\boldsymbol{\phi }}}\,({f}_{{\boldsymbol{\theta }}}\,({\bf{x}},{\bf{z}},t),t)]\}+\lambda \,{{ \mathcal L }}_{\mathrm{ROI}}.\end{array}\end{eqnarray*}

### 2.2. HyperNetwork

**Figure 2.**
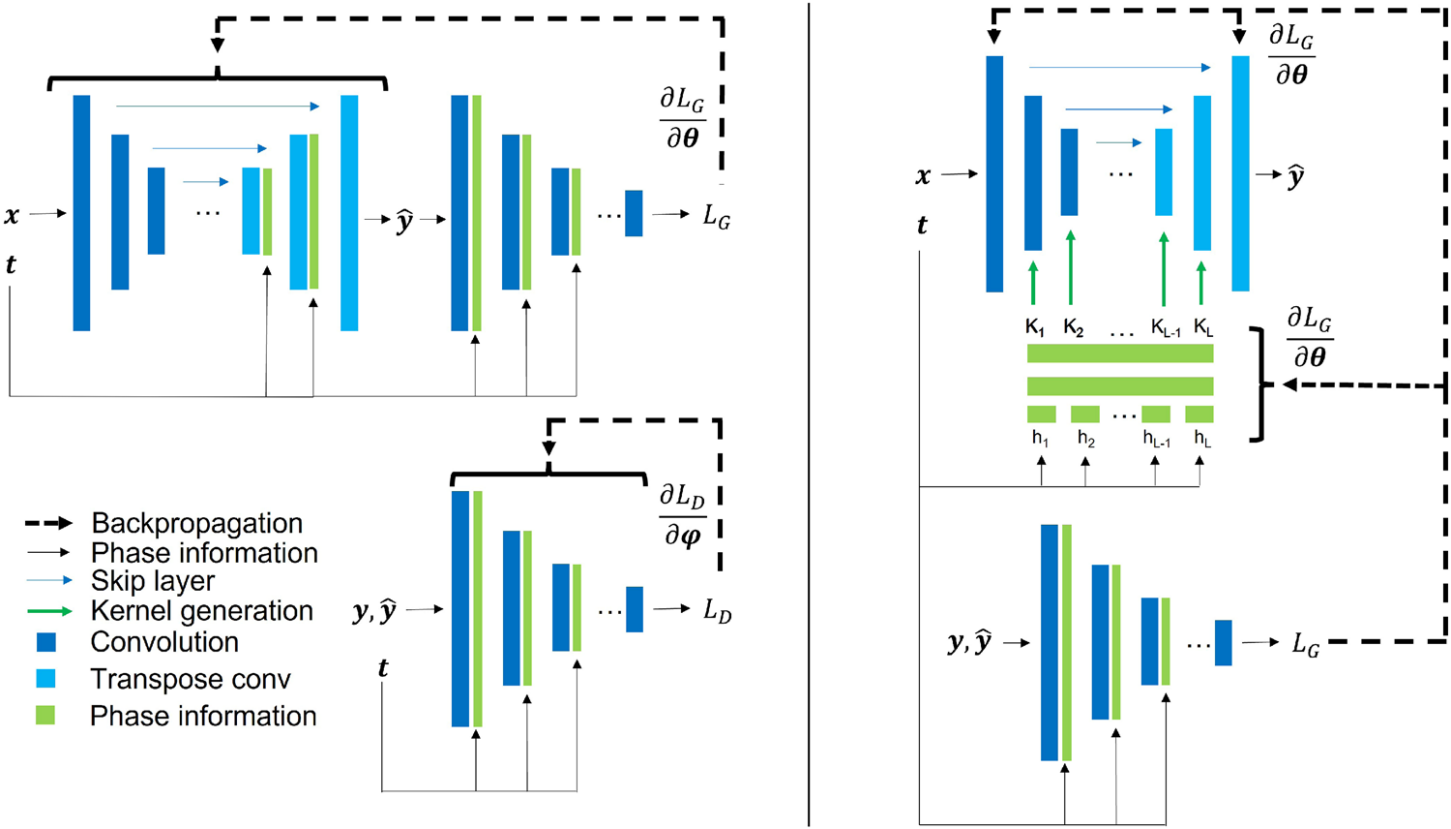
Left: training pipeline for Pix2Pix generator (top) and discriminator (bottom). Right: training pipeline for HyperPix2Pix generator.

The HyperNetwork-based variant keeps the same generator architecture, but the kernels for each layer except the first and last are generated by the HyperNetwork. This process starts with learnt embedding vectors **h**_*i*_ (corresponding to each convolutional kernel **K**_*i*_) that are passed into the two-layered linear HyperNetwork. The outputs for each **h**_*i*_ are then reshaped into kernels **K**_*i*_, which make up the generators’s parameter set ***θ*** along with the trainable kernels for the first and last layer. The first and last layers along with the HyperNetwork are then updated through backpropagation. In this manner, instead of conditioning the generator directly on the phase information, the HyperNetwork treats phase information as a hyper-parameter and produces a different generator for each phase. The discriminator is identical to that of Pix2Pix. Figure 2 demonstrates the training process for Pix2Pix (left) and the generator in the HyperNetwork variant (right).

### 2.3. Phase information

The two network types outlined above accept phase information in different ways. For training, we normalise the time *t* after RCA injection to 1 for CME and 2 for NGE, while for inference *t* could take any value in the range [1, 2]. The standard Pix2Pix technique takes a conditional scalar input corresponding to *t*, which is then tiled and concatenated to the feature maps of both Pix2Pix generator decoder and discriminator, essentially conditioning the network on the data as in the equation below

\begin{eqnarray*}{\hat{{\bf{y}}}}_{t}={f}_{\theta }({\bf{x}},{\bf{z}},t).\end{eqnarray*}

The HyperNetwork instead treats phase information as a hyperparameter and scales the vectors **h**_*i*_ by *t* before they are transformed by the two layer HyperNetwork. The **K**_*i*_ from the HyperNetwork then form a parameter set of convolutional kernels that vary with time, ***θ*** (*t*). Predictions are then generated based on the desired *t*:

\begin{eqnarray*}{\hat{{\bf{y}}}}_{t}={f}_{\theta (t)}({\bf{x}},{\bf{z}}).\end{eqnarray*}

### 2.4. Receptive field

Preliminary experiments indicated that receptive field has a substantial effect on performance, with image sharpness improving down to a patch size of 64 × 64 × 64. Therefore, two versions of each network were trained to assess the effect of the receptive field on model performance—one utilised the entire image, i.e. with a full receptive field, while one operated on patches of size 64 × 64 × 64. This yielded two experiments, P2P-Full and P2P-Patch for the baseline Pix2Pix model, with HP2P-Full and HP2P-Patch for the HyperNetwork variant.

### 2.5. Statistical analysis

Statistical analysis was performed with RStudio 2021.09.2. Normally distributed data was tested using the two-tailed t-test, while for non-normal data the Kruskal-Wallis H-test and Mann-Whitney U-test were used. Non-normal confidence intervals were generated with non-parametric bootstrap (100 000 runs). All *p*-values from statistical tests underwent Bonferroni correction for multiple comparisons to maintain a family-wise error rate (*α*) of 0.05.

To assess the degree of bias between the different models and the ground truth voxel intensities, Bland–Altman analysis (Bland and Altman 1999) was employed. Bland–Altman analysis quantifies the bias between a proposed measurement technique (here, the predicted sCE voxel intensities) and a reference (the ground truth voxel intensities). The mean $\overline{I}$ of the predicted and ground truth intensities are plotted on a subject-wise basis against the difference between predicted values and ground truth Δ*I*—it is this relationship that characterises the bias, while the 95% limits of agreement (LoA) are the 1.96 standard deviations of Δ*I* around this bias. Regression can be performed to differentiate a constant bias (significant intercept with non-significant slope on t-test) and a proportional bias that changes with $\overline{I}$ (significant slope). The two values of the regression slope at its two extremes can also characterise the nature of a proportional bias. A positive bias at the lowest mean intensity for a given model with a negative bias at the highest mean intensity indicates that the model over-enhances less intense features and under-enhances more intense ones, while the converse is also true (Ho *et al* 2018).

In order to compare the techniques biases, ANCOVA with interaction is employed. First, interaction term significance is tested to ensure no significant difference between bias slopes for each model. If the models have no significant difference in bias, ANCOVA the tests for significantly different mean biases between the techniques. ANCOVA allows us to control for intensity as a covariate, which may have an effect on the mean bias if the models’ predicted outputs vary proportionally with intensity. Pairwise statistical contrast analysis (Poline and Brett 2012) is then performed to determine which models are significantly different from one another.

### 2.6. Data and pre-processing

The retrospective data were fully anonymised after approval from the local clinical governance committee, comprising pre-procedural iCT volumes from renal cryoablation cases performed by the interventional oncology service at University College London Hospital. CME and NGE phase scans made up the two types of target image, while the source image volumes were NCE scans taken before RCA administration. All volumes were of slice thickness 1 mm and downsampled to a resolution of 256 × 256 in the *x* and *y* directions with variable depth. All images were windowed to [−500, 2500] to remove noise and highly attenuating features before being normalised to [0, 1]. For the foreground regions used to weight the loss ${{ \mathcal L }}_{\mathrm{ROI}}$, the kidneys and tumour were manually segmented from the CE images, and the aorta and major tributaries were extracted using Otsu’s method in 3D Slicer (Fedorov *et al* 2012). The training dataset numbered 35 procedures from 34 subjects, 5 of which were chosen at random for use as a validation set. An additional 15 subjects were processed as a test dataset. Owing to the large image volume size, sub-volumes of depth 64 were randomly sampled during the training process when training on whole images. To reconstruct the full-sized images from the patch-based networks, the models were passed over the input image with stride 16 and the overlapping patches were averaged. For testing on out-of-distribution data, two intra-procedural images containing needles and a post-procedural image featuring the ice ball following cryotherapy were sampled from the test subjects and underwent the same pre-processing. The initial contrast enhancement has faded by this point, and the images contain previously unseen needles and artefacts.

### 2.7. Network training

The networks were trained using Tensorflow 2.3 on a Nvidia P5000 16Gb graphics card with minibatch size 1. The Adam optimiser was used (*β*_1_ = 0.5, *β*_2_ = 0.999) with He initialisation (He *et al* 2016). A differentiable data augmentation strategy allowing back-propagation through the generator (Zhao *et al* 2020) was employed, that applies random translation, cut-out and intensity/contrast alteration to both input images and generated images seen by the discriminator.

### 2.8. Evaluation

The output patches from the patch-based models were recombined into a full-sized image by running the model over the image with stride 16 and averaging the output from overlapping patches, before then converting the intensities back into Hounsfield units (HU). All predicted image volumes underwent visual examination and were evaluated using mean squared error (MSE), peak signal-to-noise ratio (pSNR) and structural similarity index measure (SSIM), both globally and restricted to the foregound ROIs. For further evaluation of the contrast-enhanced ROIs, 5 equally-spaced slices containing the aorta (Ao), renal cortex (Co), medulla (Md) and tumour (Tu), except in one subject where the tumour could not be confidently identified, were segmented. The ROIs are hereafter referred to by abbreviations with the phase as a subscript, e.g. Md_CME_ for medulla CME phase and Tu_NGE_ for aorta NGE phase. To generate arbitrary sCE phases, the time input to the models was adjusted between 1 and 2 (corresponding to 35s CME and 80s NGE)—3 additional phases were generated corresponding to 46s, 57s and 69s.

Segmentation was used as the downstream task to examine whether it was feasible to generate an optimal sCE phase. For this purpose, a U-Net (Ronneberger *et al* 2015) was trained to segment tumours from sCE images generated by the models during the testing phase. The segmentation training set comprised images from 5 sCE phases (35 s, 46 s, 57 s, 69 s, 80 s) for each model, which were converted into 2D 64 × 64 patches and used for training using minibatches of size 64 and learning rate 10^−3^. The training split contained 247 slices from 10 of the 14 test subjects which contained an identifiable tumour, along with ground truth CME and NGE images (241 total). The segmentation testing split contained slices from the 4 remaining test subjects, totalling 91 model output patches and 95 ground truth patches.

### 2.9. Hyper-parameter tuning

Following random search hyper-parameter tuning, the optimal generator and discriminator architectures for both experiments utilised 7 and 3 layers, and 32 and 16 first layer channels respectively. The optimal Pix2Pix model had discriminator and generator learning rates of 2.0 × 10^−5^ and 3.5 × 10^−4^, and *λ* and *μ* of 720 and 0.1, while these values were 1.0 × 10^−4^, 5.6 × 10^−4^, 630 and 0.1 for HyperPix2Pix.

## 3. Results

### 3.1. Image quality

The median values for the three image quality metrics (across the entire image) are shown for each model in table 1 along with their bootstrap-derived confidence intervals. Both patch-based methods performed significantly better than their full-sized counterparts on U-test in terms of both MSE and pSNR (Pix2Pix CME *p* ≤ 0.00001 and NGE *p* ≤ 0.00001, HyperNetwork CME *p* ≤ 0.001 and NGE *p* ≤ 0.001). This effect did not extend to SSIM and there was no significant difference between HyperNetwork and non-HyperNetwork techniques. In addition, we report the same metrics restricted to the image foreground in table 2, i.e. all 4 ROIs—we see that HP2P-Patch outperforms P2P-Patch in SSIM for the NGE phase (*p* < 0.008).

**Table 1.** Image quality—global.

Model	NCE → CME	NCE → NGE
MSE		

P2P-Full	14469 [13623, 15636]	13566 [11869, 14958]
P2P-Patch	**8374 [7190, 9713]**	**8011 [6390, 10172]**
HP2P-Full	12836 [12277, 13571]	12582 [11509, 14828]
HP2P-Patch	**8154 [6939, 10319]**	**7589 [5916, 9471]**

pSNR		

P2P-Full	54.72 [54.17, 54.97]	55.00 [54.82, 55.52]
P2P-Patch	**57.10 [56.58, 57.67]**	**57.29 [55.93, 58.09]**
HP2P-Full	55.25 [54.98, 55.85]	55.33 [54.48, 55.69]
HP2P-Patch	**57.22 [55.88, 57.82]**	**57.53 [56.40, 58.39]**

SSIM		

P2P-Full	0.9938 [0.9924, 0.9959]	0.9947 [0.9939, 0.9977]
P2P-Patch	0.9937 [0.9924, 0.9953]	0.9941 [0.9921, 0.9958]
HP2P-Full	0.9934 [0.9920, 0.9947]	0.9936 [0.9912, 0.9949]
HP2P-Patch	0.9949 [0.9933, 0.9974]	0.9949 [0.9930, 0.9972]

**Bold**: significance for patch-based vs full-sized.

Reported values: median [95% CI].

**Table 2.** Image quality—foreground.

Model	NCE → CME	NCE → NGE
MSE		

P2P-Full	11005 [4768, 13232]	2751 [1280, 3569]
P2P-Patch	12082 [6689, 15505]	3666 [2695, 5020]
HP2P-Full	11657 [7139, 15979]	3434 [2763, 4802]
HP2P-Patch	10951 [5151, 15375]	2339 [1687, 3146]

pSNR		

P2P-Full	55.91 [55.03, 57.86]	61.94 [60.40, 63.80]
P2P-Patch	55.51 [54.06, 57.11]	60.69 [58.69, 61.71]
HP2P-Full	55.66 [54.63, 57.09]	60.97 [58.76, 62.53]
HP2P-Patch	55.93 [53.69, 57.78]	62.64 [60.80, 64.58]

SSIM		

P2P-Full	0.9887 [0.9873, 0.9994]	0.9970 [0.9958, 0.9976]
P2P-Patch	0.9890 [0.9860, 1.0000]	**0.9949 [0.9930, 0.9976]**
HP2P-Full	0.9868 [0.9832, 0.9943]	0.9961 [0.9946, 0.9969]
HP2P-Patch	0.9903 [0.9862, 1.0000]	**0.9975 [0.9962, 0.9986]**

**Bold**: pairwise significance.

Reported values: median [95% CI].

### 3.2. Quantitative analysis

**Figure 3.**
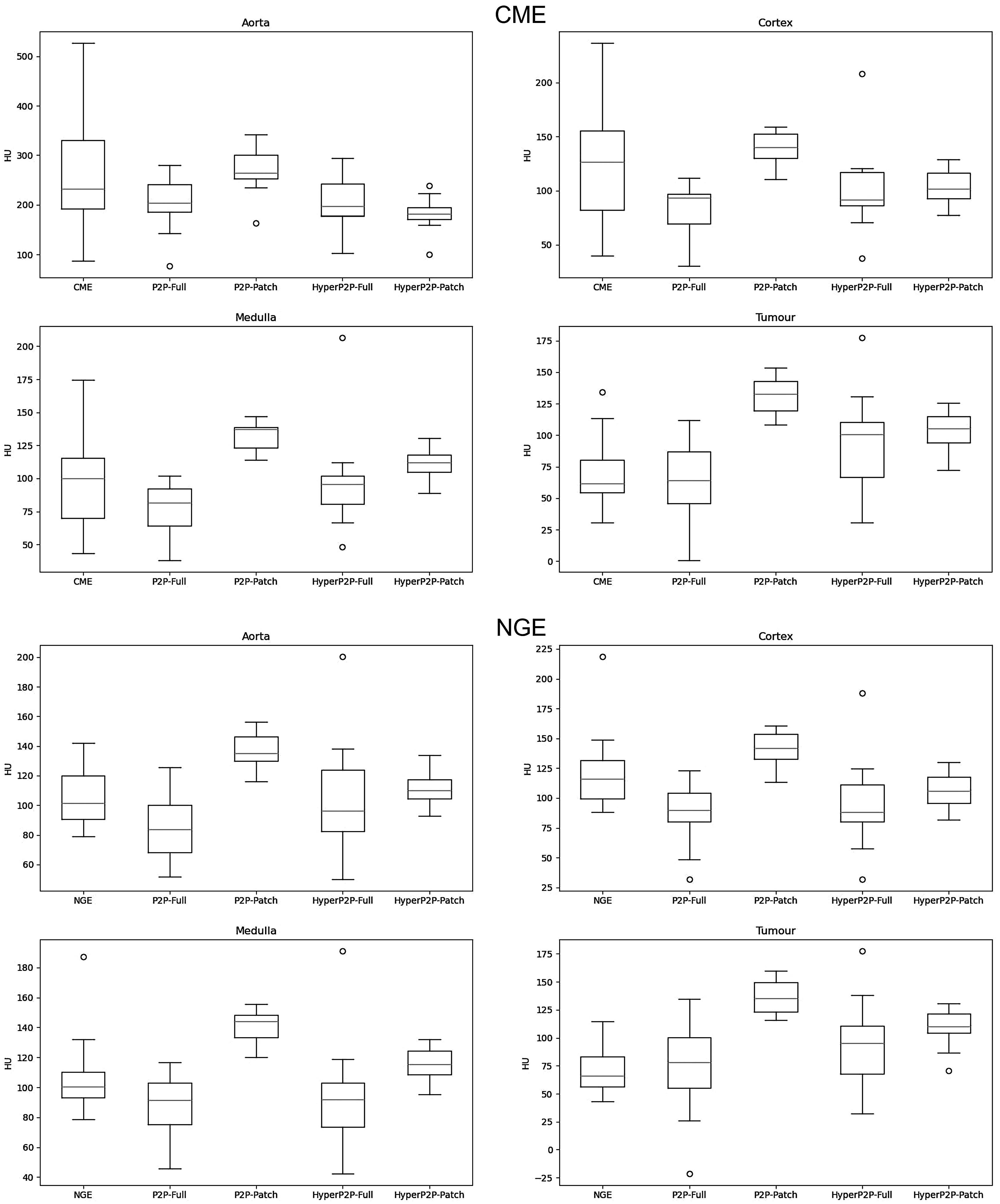
ROI intensities for generated CME (top) and NGE (bottom) images.

Figure 3 shows the predicted ROI intensities for each network, along with the ground truth values. The predictions by P2P-Patch were brighter than other models, reaching significance in all cases except for HP2P-Full’s aorta predictions in the CME phase, while HP2P-Patch was brighter than P2P-Full in Tu_CME_ and in medulla for both phases (all *p* < 0.001). There were no significant differences between the two HyperNetwork-based models. Table 3 shows the biases between predicted intensities and ground truth for each of the models. Both patch-based methods had a significantly positive regression slope on t-test (*p* < 0.001) for aorta, cortex and medulla in CME images, reflecting a proportional bias for these techniques (indicating that the methods’ bias is dependent on the intensity in these strongly-enhancing ROIs). The regression intercept term gives us the mean bias of each model. P2P-Patch over-enhances medulla and tumour in both phases, as well as Ao_NGE_. HP2P-Patch under-enhances Ao_CME_ and over-enhances Tu_NGE_, while P2P-Full under-enhances Co_CME_. HP2P-Full has no significant bias in any region.

**Table 3.** ROI biases and slopes.

Model	CME bias (HU)	NGE bias (HU)
Aorta		

P2P-Full	−53 [±127] (−0.94)	−21 [±45] (0.22)
P2P-Patch	14 [±123] (−**1.16**)	**30** [±25] (−0.57)
HP2P-Full	−49 [±138] (−1.01)	−2 [±71] (1.23)
HP2P-Patch	−**74** [±105] (−**1.45**)	5 [±32] (−0.80)

Cortex		

P2P-Full	−**42** [±66] (−1.09)	−32 [±63] (−0.43)
P2P-Patch	15 [±54] (−**1.51**)	21 [±48] (−1.08)
HP2P-Full	−24 [±106] (−0.69)	−26 [±91] (0.11)
HP2P-Patch	−21 [±57] (−**1.76**)	−14 [±52] (−1.30)

Medulla		

P2P-Full	−24 [±52] (−1.04)	−20 [±50] (−0.39)
P2P-Patch	**31** [±34] (−**1.48**)	**33** [±32] (−1.10)
HP2P-Full	−4 [±88] (−0.32)	−13 [±74] (0.38)
HP2P-Patch	11 [±43] (−**1.67**)	8 [±38] (−1.33)

Tumour		

P2P-Full	−8 [±70] (0.24)	2 [±74] (1.29)
P2P-Patch	**61** [±47] (−1.05)	**66** [±49] (−0.55)
HP2P-Full	24 [±84] (0.54)	22 [±76] (1.36)
HP2P-Patch	34 [±56] (−1.11)	**38** [±54] (−0.53)

Data format: mean bias [95% LoA] (slope coefficient).

**Bold** indicates a significant proportional bias.

For each ROI and phase, the regression lines for each model were compared using interaction terms in the ANCOVA model—none of these were significant. The mean biases for all regions except Tu_NGE_ were significantly different when adjusting for $\overline{I}$ as a covariate. On pairwise testing, P2P-Full, HP2P-Full and HP2P-Patch all had a significantly more negative bias for Ao_CME_ and Co_CME_ than P2P-Patch, while P2P-Patch had a significantly positive bias compared to both full receptive field methods in medulla (both phases) and P2P-Full in Tu_CME_.

### 3.3. Qualitative results

**Figure 4.**
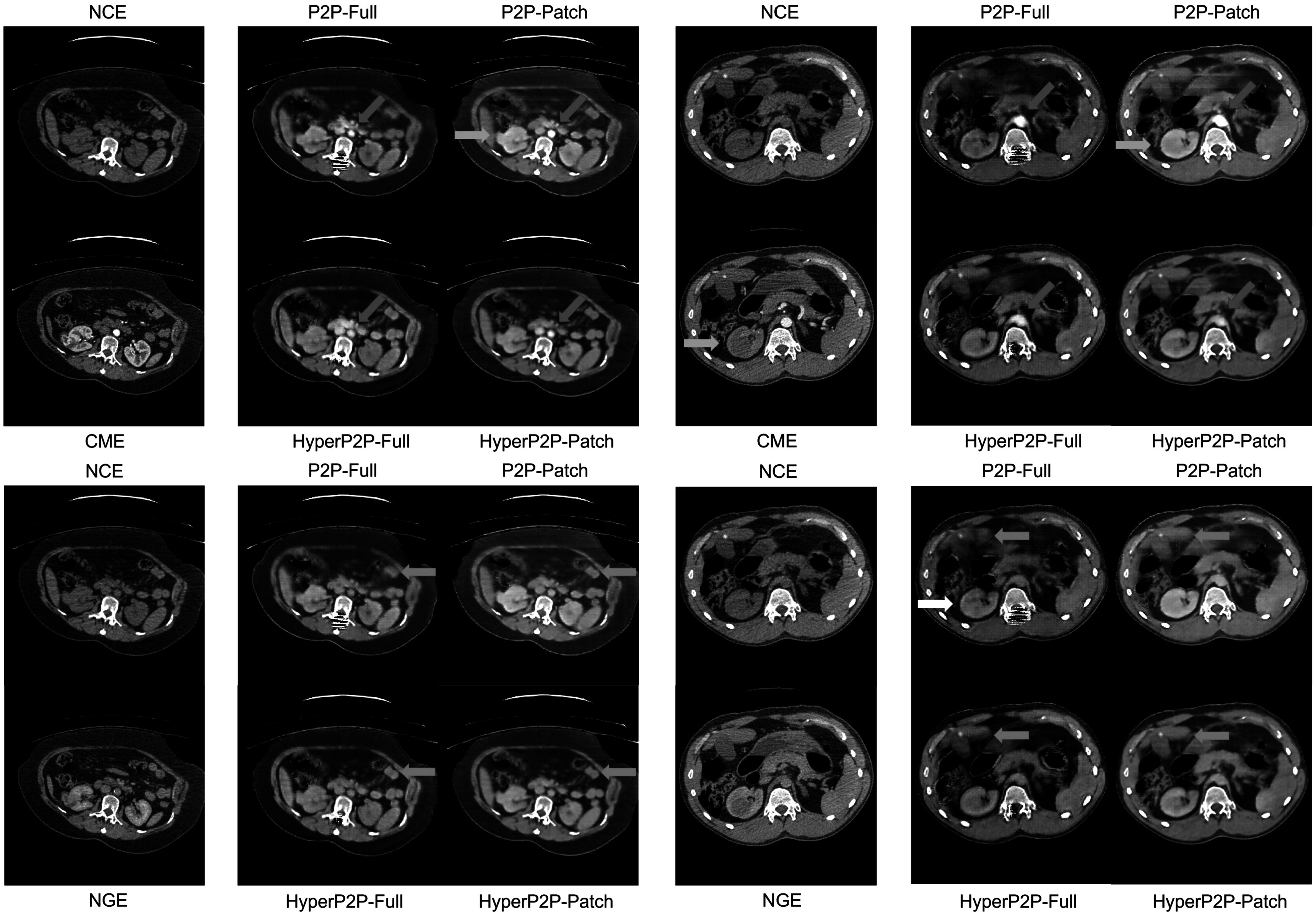
Example predictions for corticomedullary contrast (top block) and nephrogenic contrast (bottom block).

Figure 4 and Supplemental figure S2 (online) show examples of the predicted CME and NGE phases compared with the NCE input images and ground truths. In keeping with the lower MSE and higher pSNR between patch-based and full-receptive field methods seen in table 1, image quality appears slightly better in the central regions of the patch-based predictions, with sharper features and better-defined contrast around the aorta. All models enhance the aorta and kidneys in CME, with the renal cortex appearing slightly brighter than medulla. While the intensity of the aorta has decreased appropriately on predicted NGE images, there is little visual difference between the renal enhancement patterns between the two phases.

The HyperNetwork-based models appear to generate dimmer contrast-enhancing ROIs than their counterparts (see red arrows, figure 4, while the background regions in some cases are sharper (blue arrows)). In addition, HP2P-Patch does not suffer from the checkerboard artefacts seen in P2P-Patch to the same extent. P2P-Patch generates bright predictions in CME images where the ground truth is poorly-enhancing (see green arrows, figures 4 and S2); however the same network also shows a tendency to over-enhance ROIs around the tumour and tumour itself (green arrows, figure 4), in keeping with the positive bias seen in table 3. Regarding the differences between full receptive field and patch-based techniques, we see in figure 4 (white arrow) that P2P-Full has generated greater detail in the tumour, while P2P-Patch has overly smoothed this region. In figure S2 (white arrows), while the predicted contrast-enhancement pattern is different from the ground truth, both full receptive field models have added greater detail to the prediction compared to the the patch-based methods.

### 3.4. Peri-procedural contrast enhancement

**Figure 5.**
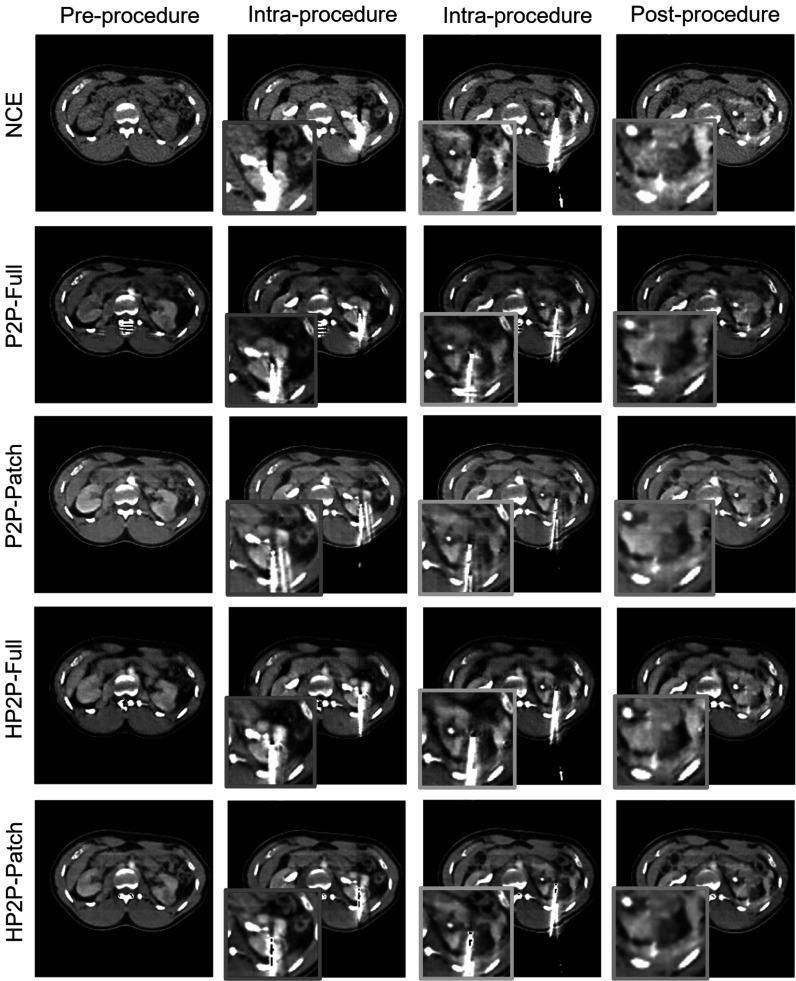
CME images from before, during and after procedure. Red and green insets: in-painting of photon starvation artefact; blue inset: ice ball contrast enhancement.

**Figure 6.**
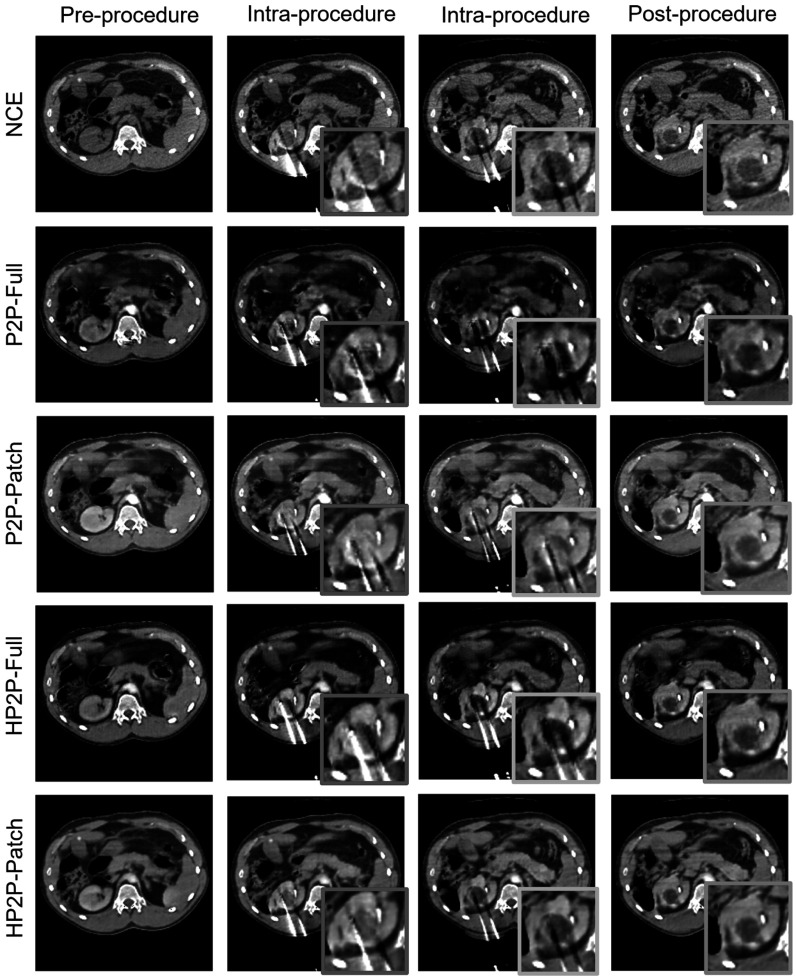
CME images from before, during and after procedure. Red and green insets: variable visualisation of tumour/ice ball; blue inset: ice ball contrast enhancement.

Figures 5 and 6 show the output of the models when applied to unseen data from later time points in the procedure: two intra-procedural, one post-procedural, and the NCE image for comparison. The networks manage to perform sCE, despite distracting features such as needles and ice ball formation not previously encountered during training. In addition, all networks in-paint the photon starvation artefact caused by the needles (figure 5, red and green insets), although we see that P2P-Patch has erroneously added needles from neighbouring slices (red inset). Tumour and ice ball visualisation is more variable, with P2P-Patch over-enhancing the tumour in the intra-procedural images (figure 6, green and red insets) compared to the other networks (particularly HP2P-Full). The two HyperNetwork-based models perform better at improving ice ball visualisation (by lowering the voxel intensity in this ROI) in the post-procedural images (figures 5 and 6, blue inset), behaviour also seen from P2P-Full in figure 6. P2P-Patch once again subtly over-enhanced the ice ball in these images compared to the other networks. All networks have also reduced the streaking artefacts seen around the needles, improving their visualisation against the background image.

### 3.5. Contrast phase interpolation

**Figure 7.**
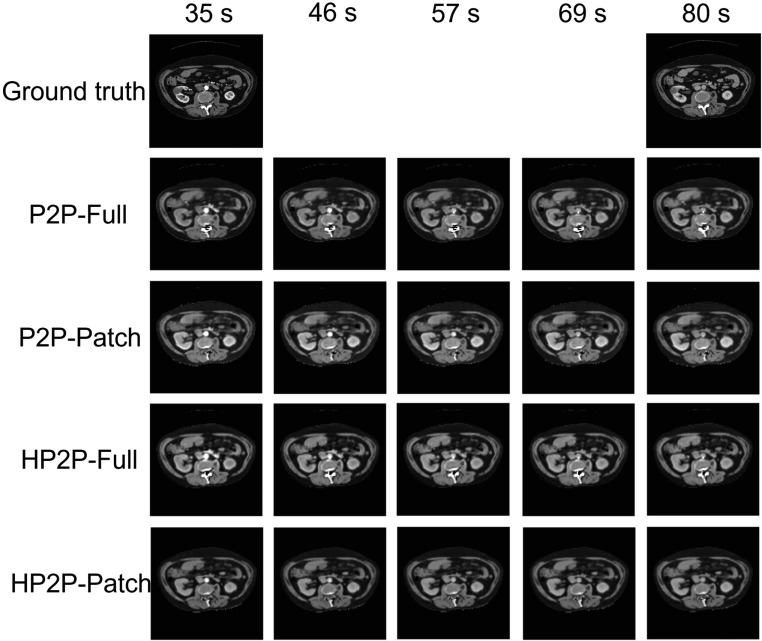
Interpolated contrast phases between CME and NGE, with ground truth phases for comparison.

Figure 7 shows the interpolation between the CME and NGE phases for each of the networks. All four networks interpolate the intensities in the aorta between CME and NGE in a realistic manner, with little observable difference between individual performance. Outside of the aorta, however, there is little difference between the predicted intensities at different time points in the renal and tumour ROIs. This is confirmed statistically on H-test, where there were statistically significant (*p* < 0.002) differences in the intensity of the aorta over time for all models, but no significance in the other ROIs. This matches the situation seen in the ground truth phases, where significance between CME and NGE was seen on U-test in the aorta (*p* < 0.002), but not in the other ROIs.

### 3.6. Task-based evaluation

Table 4 shows results of a 2D U-Net trained on images predicted by each sCE network along with the ground truth phases for comparison. The generated time-points range between CME (35s) and NGE (80 s), created by interpolating between the two time inputs provided during training. All models except HP2P-Patch had significantly different Sø renson-Dice scores depending on the phase generated, with non-HyperNetwork model performances being higher at shorter time points, and HP2P-Full performance being higher at later points. The optimal time for P2P-Patch was 35 s (0.559), while the full receptive field networks had optimal time-points between 57 and 69 s (0.423 and 0.444 respectively). HP2P-Patch consistently generated Sø renson-Dice scores between 0.386 and 0.464, with no significant performance decrease. The best and worst performing time-points were all significantly different between models on H-test. Of the best-performing contrast time-points, P2P-Patch had the highest Sø renson-Dice score of 0.559 (*p* < 0.05), while of the lowest-performing, HP2P-Patch had the highest score of 0.386 (*p* < 0.05).

**Table 4.** Dice scores (median).

Time (s)	Ground truth	P2P-Full	P2P-Patch	HP2P-Full	HP2P-Patch
35	0.436	0.417	**0.559**	0.182	0.444
46	–	0.351	0.400	0.235	0.386
57	–	**0.423**	0.390	0.237	0.419
69	–	0.225	0.316	**0.444**	0.464
80	0.650	0.000	0.287	0.436	0.438

**Bold**: most accurate prediction by each model (significant on H-test).

### 3.7. Summary of results

The patch-based methods had a significantly better MSE and pSNR, with slightly better visual image quality in central regions. However they did tend to over-smooth regions containing tumour (with a positive bias), while the full receptive field models retained some detail in these areas. The HyperNetwork-based models typically generated dimmer ROIs, but appeared to create visually sharper background features—these include vulnerable organs such as bowel that should be clearly visible during needle insertion. HP2P-Patch also exhibited fewer checkerboard artefacts than P2P-Patch and outperformed this technique on ROI SSIM in the NGE phase. Both patch-based techniques had less variation (as reflected in their proportional bias) than ground truth in 3 CME ROIs, while P2P-Patch was typically the most strongly-enhancing network, to the extent of over-enhancing some regions (particularly medulla and tumour), and HP2P-Full had no significant bias in any region or phase.

All networks were able to generate contrast in unseen data containing previously unseen features, and improved visualisation of the needle and regions obscured by photon starvation. The HyperNetwork-based models increased the contrast of the tumour/ice ball well, while P2P-Patch over-enhanced this region (in keeping with its tendency towards brightly enhancing ROIs) and appeared to merge features from neighbouring slices. Lastly, the segmentation performance on images from all but the patch-based HyperNetwork varied according to the phase generated, with P2P-Patch having the best performing predictions when limited to one phase, and with HP2P-Patch exhibiting no significant performance decrease across all time points.

## 4. Discussion

We have introduced the use of HyperNetworks to perform multi-phase synthetic contrast enhancement and have compared their performance to that of a standard GAN-based technique. Additionally, we have compared the performance of networks performing sCE with an entire image as the the receptive field, versus those acting in a patch-wise manner.

The first hypothesis to be addressed is whether a Pix2Pix using a HyperNetwork is feasible for generating sCE images compared with a standard Pix2Pix. In terms of global image quality metrics there was no significant difference, but the HyperNetwork models did appear to generate sharper background detail and the checkerboard-type artefacts seen in P2P-Patch were less noticeable in HP2P-Patch, with a statistically significant improvement in ROI-based SSIM (NGE phase). The patch-based HyperNetwork model appears to cause less of a constant bias in medulla and tumour than its standard counterpart, while the full receptive field HyperNetwork model had no significant bias at all, indicating that HyperNetworks offer competitive performance with respect to standard neural networks. They could potentially be of use in situations where foregound ROI image quality, lack of artefacts and low bias in target regions are prioritised over intense contrast enhancement of vascular anatomy.

Regarding the second hypothesis—using image patches results in better performance—the patch-based methods offered a significantly better MSE and pSNR compared to the models using a full receptive field, as well as generating sharper detail in the central region around the aorta. The patch-based models retained the same number of parameters as the full receptive field models, while operating on a smaller region. In addition, iCT image pairings typically contain misalignment owing to soft tissue deformation and patient repositioning over the course of the procedure. The use of a smaller receptive field likely reduces the impact on the training process of distracting alignment errors elsewhere in the image, but this comes at the cost of a positive bias within the tumour region resulting in a loss of detail. The full receptive field techniques, in contrast, generate greater detail within the tumour.

The patch-based standard Pix2Pix generated the highest ROI intensities of all models, exceeding the ground truth in some cases, and both patch-based methods also demonstrated a lower variation in intensities with respect to the ground truth in some CME images. ‘Over-enhancing’ certain ROIs is not necessarily problematic, depending on the application. The more intense enhancement makes ROIs such as aorta easier to visualise, but this comes at the expense of over-enhancing tumours, which may be unwanted when trying to discern the tumour against the background. A model such as this would be more useful for identifying vascular anatomy for interventional planning when the tumour location is already known. HP2P-Full is the only network with no significant proportional or constant bias across all regions and both phases. While we cannot, based on this alone, accept the hypothesis that there is no bias for this model, this would benefit from further investigation, for instance in a blinded reader study. A similar caveat regarding the use case applies to the increased tumour detail seen in the full receptive field predictions in figure S2—while this may be beneficial in some applications, caution must be taken regarding the false positive enhancement of nearby structures.

All networks exhibit robust performance when testing on out-of-distribution data acquired during the iCT procedure and immediately afterwards, and are able to enhance the ROIs despite the previously unseen features in these images, although P2P-Patch did tend towards over-enhancing the tumour, in keeping with the bias seen in table 3. Despite the unclear mechanism for learning contrast enhancement patterns, sCE generation appears to have some utility which extends to intra- and post-procedural images. As the tumour becomes more visible during the procedure owing to the residual contrast in the washout phase and formation of an ice ball, the models enhance this contrast further, resulting in improved visualisation of these clinically relevant structures. This shows promise in enhancing the contrast not just of regions that are highly enhancing on RCA administration, but other regions such as ice balls that are of interventional importance such as treatment monitoring. Additionally, all networks in-painted regions of photon starvation and performed de-noising of needle artefacts (although P2P-Patch did add features from adjacent slices).

The last hypothesis to be tested concerned the ability to generate variable contrast phases to be used for a downstream task. While all networks generate a physically realistic decline in intensity in the aorta when interpolating between CME and NGE, the other ROIs do not show an significant change for any model. Of note is the fact that there is also no significant difference in the ground truth intensities within these ROIs. The visual difference between the two phases is driven by the change in contrast enhancement pattern as contrast agent moves from the vasculature to the nephrons rather than the change in the overall intensity of these areas. The *L* ^1^-norm—which drives supervision in the foreground ROIs—measures the mean intensity of these ROIs, and therefore generates no signal for the networks to change intensity over time to a significant degree. Future work should examine ways to improve the subject-specific contrast enhancement patterns in these ROIs by improving the use of the adversarial-based loss rather than the *L* ^1^-norm.

All networks were able to generate an interpolated phase that achieved Sø renson-Dice scores competitive with that of the ground truth CME phase. Not all interpolated time-points were suitable however, with non-HyperNetwork model performance decreasing after 69 s, and highest HP2P-Full performance at 69 s and 80 s. HP2P-Patch was able to generalise contrast enhancement to multiple arbitrary phases, with no significant decrease in performance at any time.

A limitation to this pilot study is the lack of ground truth for directly evaluating a continuous range of contrast phases. Future studies could therefore include an array of downstream tasks to robustly tune the optimal contrast phase in addition to the human readability studies mentioned.

## 5. Conclusions

In this paper, we compared two frameworks for generalising the problem of synthetic contrast enhancement to multiple phases, as well as investigating the effect of receptive field on model performance. The results indicate that the use of a HyperNetwork (i.e. treating phase information as a hyper-parameter) is feasible, with a visual improvement in the detail of background features, consistent quantitative performance across all regions in the full receptive field case, and improved foreground image quality in the patch-based case. Patch-based techniques offered significantly better image quality in the central regions around the aorta, with Patch-P2P generating the most intense vascular contrast. Meanwhile, the full receptive field models provided visually better detail of tumour and some background features, with a lower bias in tumour-adjacent ROIs. Human readability studies are suggested as a future research direction to further examine the trade-offs between models in different clinical applications. Furthermore, we have shown that all models are robust enough to generalise to unseen intra-procedural data, while also improving needle artefacts and visualisation of non contrast-enhancing regions, potentially allowing future exploration of multi-task problems in this application. Lastly, the segmentation results show that there is a potential use for generating arbitrary contrast phases to optimise downstream clinical tasks. Considering the use case is advisable when deciding on a suitable model. If consistently bright intensity and high performance on generating images of one contrast phase for segmentations is required, P2P-Patch performs best. However if quantitative accuracy and consistency is more important, HP2P-Full had a smaller bias in the regions or phases over-enhanced by P2P-Patch, while HP2P-Patch was able to generate images suitable for segmentation across all time points, indicating that convolutional kernel interpolation offers some advantages in modelling contrast changes over time.

## Data Availability

The data cannot be made publicly available upon publication because they contain sensitive personal information. The data that support the findings of this study are available upon reasonable request from the authors.

## Conflicts of interest

Mark Pinnock, Yipeng Hu, Steve Bandula and Dean Barratt have no relevant financial or non-financial interests to disclose.
